# Depression and the relationship between sleep disturbances, nightmares, and suicidal ideation in treatment-seeking Canadian Armed Forces members and veterans

**DOI:** 10.1186/s12888-018-1782-z

**Published:** 2018-06-19

**Authors:** J. Don Richardson, Lisa King, Kate St. Cyr, Philippe Shnaider, Maya L. Roth, Felicia Ketcheson, Ken Balderson, Jon D. Elhai

**Affiliations:** 1 Parkwood Institute Operational Stress Injury Clinic, London, ON Canada; 2 Department of Psychiatry, Western University, London, ON Canada; 3 Department of Psychiatry and Behavioural Neurosciences, McMaster University, Hamilton, ON Canada; 4 Anxiety Treatment and Research Centre, St. Joseph’s Healthcare Hamilton, Hamilton, ON Canada; 5 Department of Graduate Studies, Ryerson University, Toronto, ON Canada; 6 Department of Psychiatry, University of Toronto, Toronto, ON Canada; 7 Departments of Psychology and Psychiatry, University of Toledo, Toledo, OH USA

**Keywords:** Major depressive disorder, Suicide, Posttraumatic stress disorder, Insomnia, Nightmares, Canadian armed forces

## Abstract

**Background:**

Research on the relationship between insomnia and nightmares, and suicidal ideation (SI) has produced variable findings, especially with regard to military samples. This study investigates whether depression mediated the relationship between: 1) sleep disturbances and SI, and 2) trauma-related nightmares and SI, in a sample of treatment-seeking Canadian Armed Forces (CAF) personnel and veterans (*N* = 663).

**Method:**

Regression analyses were used to investigate associations between sleep disturbances or trauma-related nightmares and SI while controlling for depressive symptom severity, posttraumatic stress disorder (PTSD) symptom severity, anxiety symptom severity, and alcohol use severity. Bootstrapped resampling analyses were used to investigate the mediating effect of depression.

**Results:**

Approximately two-thirds of the sample (68%; *N* = 400) endorsed sleep disturbances and 88% (*N* = 516) reported experiencing trauma-related nightmares. Although sleep disturbances and trauma-related nightmares were both significantly associated with SI on their own, these relationships were no longer significant when other psychiatric conditions were included in the models. Instead, depressive symptom severity emerged as the only variable significantly associated with SI in both equations. Bootstrap resampling analyses confirmed a significant mediating role of depression for sleep disturbances.

**Conclusions:**

The findings suggest that sleep disturbances and trauma-related nightmares are associated with SI as a function of depressive symptoms in treatment-seeking CAF personnel and veterans. Treating depression in patients who present with sleep difficulties may subsequently help mitigate suicide risk.

## Background

Sleep problems such as insomnia, nightmares, and poor sleep quality are frequently reported among military members and veterans [1]. Recent research employing data from the Deployment Life Study [2] reported that nearly half of interviewed US service members (48.6%) exceeded clinically-significant thresholds for sleep disturbances [1]. In a sample of veterans returning from Iraq or Afghanistan, Plumb and colleagues [3] reported that 89.1% met the cut off score for “poor sleep” using the Pittsburgh Sleep Quality Index. Similarly, high rates of sleep problems were found among a sample of Canadian veterans seeking treatment for mental-health conditions, with reported rates as high as 87% for trouble falling asleep, and 68% for experiencing nightmares [4]. Insomnia often serves as the initial reason military personnel seek medical treatment, and remains one of the most frequently reported complaints of treatment-seeking military members and veterans [5, 6].

This finding is largely unsurprising, given both the physical and emotional demands of an occupation in the military, and that sleep problems are a core symptom of numerous psychiatric disorders. For example, sleep disturbances are symptoms of both posttraumatic stress disorder (PTSD) and major depressive disorder (MDD) [7]. In one study, veterans with PTSD reported more impaired sleep as measured by the Pittsburgh Sleep Quality Inventory than veterans without PTSD [8]. Additionally, numerous research studies have identified a relationship between sleep-related difficulties and suicidal ideation (SI), suicide attempts, and completed suicide in both military and civilian populations [9–21], though the nature of the relationship between psychiatric conditions, sleep difficulties, and SI is complex and not fully understood.

Some research suggests that the relationship between sleep disturbances and SI is best explained by the presence of psychiatric diagnoses (i.e., there is an indirect effect of sleep disturbance on SI). For example, Bernert and colleagues [11] and Liu [22] both found that the relationship between nightmares and suicidal behaviours was statistically significant even when accounting for depression. Richardson and colleagues [4] found that, when controlling for probable diagnoses of PTSD, MDD, generalized anxiety disorder (GAD), and alcohol use disorder (AUD) in a sample of treatment-seeking CAF members and veterans, only probable MDD emerged as a significant predictor of SI. Similarly, Bryan and colleagues [23] found that depression significantly mediated the relationship between insomnia severity and suicide risk in three clinical samples of military personnel. In contrast, evidence suggesting that sleep disturbances have a direct effect on suicidal behaviours also exists. This position asserts that the relationship between sleep disturbances and SI is not accounted for by psychiatric conditions, but that sleep-related difficulties represent an independent risk factor for suicidal behaviours. For example, Ribeiro and colleagues [19] found that insomnia symptoms outperformed a number of other variables such as depression, hopelessness, PTSD, anxiety, and alcohol abuse in predicting SI using a cross-sectional and longitudinal design. After adjusting for mental health and substance use symptoms, Pigeon and colleagues [24] found that time to completed suicide was influenced by sleep problems, such that veterans who complained of sleep difficulties died by suicide sooner than those who did not.

As the number of military personnel suffering from psychiatric conditions such as PTSD and MDD [25–27], and rates of completed suicide [28–32] continue to rise, not only is it important to discern risk factors for suicidal behaviours, but also how these risk factors exert their effects (i.e., directly or indirectly). Such research is critical in order to better inform treatment initiatives, especially given the complexity of treating military members presenting with comorbid mental health conditions [33]. If sleep problems directly impact SI beyond the effect of psychiatric conditions, specific and targeted interventions for sleep may, in turn, reduce risk of SI. However, if the relationship between sleep and SI is mediated by psychiatric diagnoses, such as depression, targeting only specific sleep symptoms may provide little in the way of attenuating risk of SI.

Given the mixed results obtained in previous research, the aim of the current study was to further investigate the relationship between sleep disturbances (i.e., difficulty falling or staying asleep), trauma-related nightmares, and SI in a sample of treatment-seeking CAF members and veterans.

## Method

### Participants and procedure

Participants were treatment-seeking CAF members and veterans presenting to a specialized outpatient mental health clinic for military service-related psychiatric conditions. All measures were administered as part of a standardized intake protocol. Informed consent to use intake data for research and program evaluation was obtained from participants at the time of their initial clinical assessment. Once consent was obtained, data were de-identified and stored in an electronic database. The present study used previously-collected intake data from 663 CAF members and veterans presenting to the clinic between April 2004 and September 2014. Individuals who were referred to the clinic more than one time during this period were included in the study only once (e.g., any subsequent referrals were not included). Ethical approval was received from Western University and relevant hospital review boards.

### Measures

The Patient Health Questionnaire (PHQ) is a self-administered version of the Primary Care Evaluation of Mental Disorders (PRIME-MD) used to evaluate mood, anxiety, and somatoform disorders [34]. Symptoms of depression were measured using the PHQ-9, a nine-item measure used to evaluate depressive symptom severity based on DSM-IV diagnostic criteria. Respondents indicated how frequently they experienced each symptom in the past two weeks on a scale ranging from 0 = “Not at all” to 3 = “Nearly every day.” Responses were summed to provide a total score ranging from 0 to 27, where higher scores are indicative of greater depressive symptom severity [35].

SI was measured using a single item from the PHQ-9: “Thoughts that you would be better off dead or hurting yourself in some way.” This item has been used as a measure of SI in previous research [4, 36]. To reduce the potential for artificially inflated relationships among depressive symptom severity, sleep disturbances, and SI, two items, one assessing SI, and the other assessing sleep disturbances, were removed from PHQ-9 depressive symptom severity scores. Internal consistency of the PHQ-9 with and without these items was good (Cronbach’s alpha = 0.89 and 0.87, respectively).

PTSD symptom severity was measured using the PTSD Checklist – Military version (PCL-M) [37]. The PCL-M is a 17-item, self-administered questionnaire measuring PTSD symptom severity specific to a military-related trauma. Respondents were asked to indicate how much they had been affected by each symptom over the past month using a scale ranging from 1 = “Not at all” to 5 = “Extremely.” Responses were summed to provide a total score ranging from 17 to 85, where higher scores indicate greater PTSD symptom severity. Consistent with previous research studies and recommendations [37], a cut-off score of 50 was used to establish the presence of “probable” PTSD.

Sleep disturbances and trauma-related nightmares were measured using individual items from the PCL-M. The item assessing sleep disturbances asked participants to indicate how often they had trouble falling and staying asleep; the item assessing nightmares asked participants to indicate how often they experienced disturbing dreams of a stressful military experience. Both of these items have been used in previous research to examine sleep disturbances and nightmares [4]. In order to reduce the potential for artificially inflating the relationship between sleep disturbances, trauma-related nightmares, and PTSD symptom severity, these items were removed from PCL-M scores. Internal consistency of the PCL-M with and without the items on sleep disturbance and nightmares was excellent (Cronbach’s alpha = 0.93 and 0.92, respectively).

Alcohol use was measured using the Alcohol Use Disorders Identification Test (AUDIT): a 10-item, self-report questionnaire identifying harmful alcohol use behaviours and dependence [38]. Responses were summed to provide a total score ranging from 0 to 40, where higher scores indicate more problematic alcohol use behaviours. A score of 8 or higher is typically used to signify a potential alcohol use problem [38, 39]. Internal consistency of the AUDIT in the current sample was good (Cronbach’s alpha = 0.88).

Anxiety symptoms were measured using the GAD-7 module of the PHQ [34]. Respondents were asked to rate how often in the past two weeks they experienced each of seven symptoms on a scale ranging from 0 = “Not at all” to 2 = “More than half the days.” Responses were summed to provide a total score ranging from 0 to 14, where higher scores indicate greater anxiety. Internal consistency of these seven items in the current sample was excellent (Cronbach’s alpha = 0.94).

### Statistical analyses

SPSS Statistics v. 23.0 (Chicago, IL) was used for all analyses. Correlations between all variables were examined. Associations between sleep disturbances and SI, and nightmares and SI, were first investigated in two steps. Step 1 examined whether each variable (sleep disturbances or nightmares) was associated with SI using univariate linear regression. In Step 2, PHQ-9, PCL-M, AUDIT, and GAD-7 severity scores were added to each model.

To test whether the effects of sleep disturbances and trauma-related nightmares on SI were mediated by psychiatric conditions, we used the bootstrapping method with bias-corrected confidence estimates proposed by Preacher and Hayes [40]. Simulation studies have shown good accuracy and power in detecting mediation effects using this approach [41]. The 95% confidence intervals (CIs) for indirect effects were examined, and 5000 bootstrap resamples were used for each. CIs that do not cross zero indicate a significant mediating effect. Age, sex, level of education, and marital status were entered into the models as covariates.

## Results

The vast majority of participants were male (*N* = 546; 91.0%), and the mean age was 44.6 years (*SD* = 13.98). Participants served an average 13.78 years (*SD* = 9.26) and had been deployed 2.55 times on average (*SD* = 9.26). Most participants were veterans of the CAF (*N* = 546; 82.4%); the remaining participants were still actively serving (*N* = 117; 17.6%). Table 1 presents the means and standard deviations for each measure, as well as frequencies for sleep disturbances, trauma-related nightmares, SI, and deployment locations for the 378 participants who were deployed.

**Table 1:** Descriptive statistics

Categorical Variable	*n*	%
Deployment locales		
Afghanistan	117	31.0
Balkan States (former Yugoslovia, Kosovo, etc.)	133	35.2
Domestic deployment	125	33.1
Africa (Somalia, Rwanda, Sierra Leone, etc).	45	11.9
Education (*N* = 574)		
Less than high school	113	19.7
Completed high school	179	31.2
Some college/university	159	27.7
Completed college/university	123	21.4
Marital status (*N* = 574)		
Married/living with partner	325	56.6
Divorced/separated	125	21.8
Single (never married)	109	19.0
Widowed	15	2.6
Sleep Disturbances (*N* = 588)		
Not at all/ a little bit	188	32.0
Moderately/quite a bit/extremely	400	68.0
Nightmares (*N* = 585)		
Not at all/a little bit	69	11.8
Moderately/quite a bit/extremely	516	88.2
Suicidal Ideation (*N* = 529)		
Not at all	290	54.8
Some days	151	28.6
More than half the days/nearly every day	88	16.6
PCL-M criteria met for probable PTSD (score > = 50; *N* = 554)	402	72.6
PHQ-9 criteria met for probable MDD (score > =10; *N* = 522)	413	79.1
Measure	Mean	*SD*
PCL-M	57.71	14.78
PHQ-9	15.85	6.60
GAD	10.06	3.40
AUDIT	8.06	8.09

*Note. PCL-M* Posttraumatic Stress Disorder Checklist – Military Version, *PHQ-9* Patient Health Questionnaire-9, *AUDIT* Alcohol Use Disorders Identification Test, *GAD* Generalized Anxiety Disorder section of the PHQ

The majority of participants (68%) indicated they had trouble falling or staying asleep at a severity of “moderately or greater,” whereas 88% of our sample indicated they experienced trauma-related nightmares at a severity of “moderately or greater.” Nearly half of participants (45.2%) endorsed experiencing SI at least some days. On average, participants had moderately severe depression and most met screening criteria for probable PTSD (see Table 1).

Correlation analyses revealed significant associations between SI and PTSD symptom severity, depressive symptom severity, trauma-related nightmares, sleep disturbances, and anxiety symptom severity. Severity of alcohol use was not significantly correlated with SI, and was thus omitted from further analyses (see Table 2).

**Table 2:** Correlations

Variable	1	2	3	4	5	6	7	8	9	10	11
1. SI		.406***	.529***	.172**	.272**	.073	.382**	.039	.052	.025	−.080
2. PCL-M			.688***	.593***	.546**	.128**	.584**	−.012	−.010	−.095*	−.064
3. PHQ-9				.345**	.520**	.033	.677**	−.031	.028	−.048	−.053
4. Nightmares					.375**	.059	.234**	.043	−.059	−.024	−.016
5. Sleep disturbances						.105*	.482**	−.027	.012	.008	−.053
6. AUDIT							.082	−.106*	−.086*	.118**	−.039
7. GAD								−.012	.031	−.081	−.052
8. Age									−.071	−.186**	−.100*
9. Sex										.070	.169**
10. Marital status											.087*
11. Level of education											

*Note. SI* Suicidal ideation, *PCL-M* Posttraumatic Stress Disorder Checklist – Military Version, *PHQ-9* Patient Health Questionnaire-9, *AUDIT* Alcohol Use Disorders Identification Test, *GAD* Generalized Anxiety Disorder section of the PHQ

*p < .05, **p < .01, ***p < .001

As expected, based on significant univariate correlations, sleep disturbances and nightmares were both significantly associated with SI. However, after adding the PTSD, depression, and anxiety symptom severity variables, neither the relationship between sleep disturbances and SI, nor nightmares and SI, remained significant. Instead, depressive symptom severity emerged as the only variable significantly associated with SI in both cases (see Table 3). Variation inflation factors did not indicate any issues with multicollinearity.

**Table 3:** Regression analyses examining associations between sleep disturbances, nightmares and suicidal ideation

Nightmares	SI	Sleep Disturbances	SI
Step 1		Step 1	
Nightmares	.312**	Sleep disturbances	.219***
Step 2		Step 2	
Nightmares	.001	Sleep disturbances	−.253
Anxiety (GAD-7)	.007	Anxiety (GAD-7)	.009
PTSD (PCL-M)	.002	PTSD (PCL-M)	.003
Depression (PHQ-9)	.083***	Depression (PHQ-9)	.084***

*Note. SI* Suicidal ideation, *PCL-M* Posttraumatic Stress Disorder Checklist – Military Version, *PHQ-9* Patient Health Questionnaire-9, *GAD* Generalized Anxiety Disorder section of the PHQ

*Note:* Presented are unstandardized beta coefficients

**p = < .01, ***p < .001

**Fig. 1:**
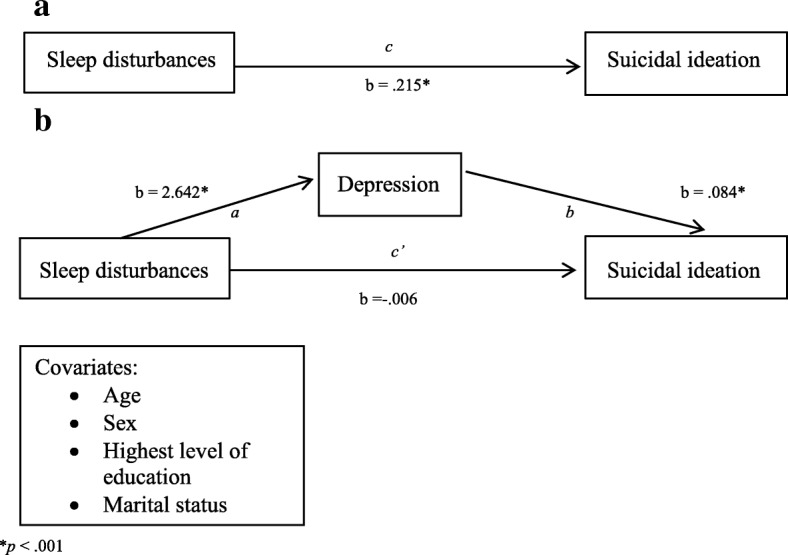
Association (**a**) and  Mediation model for sleep disturbances and suicidal ideation (**b**)

**Fig. 2:**
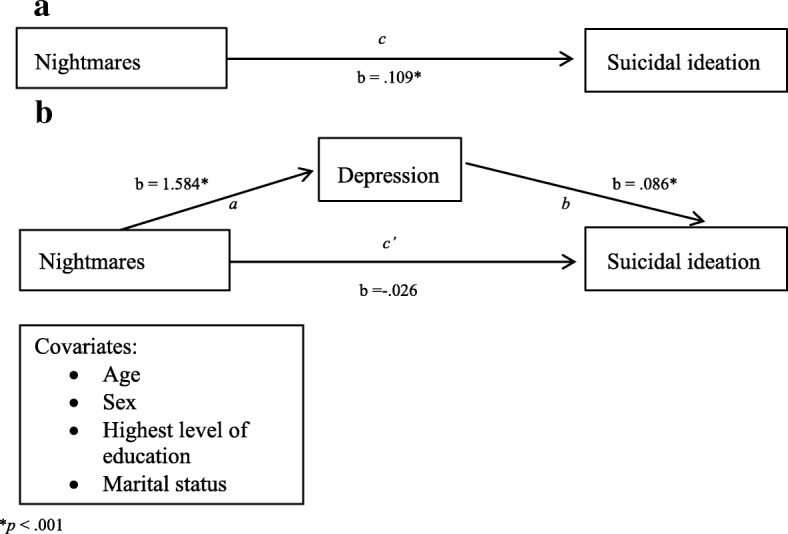
Association (**a**) and Mediation model for nightmares and suicidal ideation (**b**)

In the first model, sleep disturbances were significantly associated with SI (b = .215, SE = .035, *p* < .001). Using Preacher and Hayes’ 2004 bootstrapping method, depressive symptom severity was found to significantly mediate the relationship between sleep disturbances and SI [bootstrapped unstandardized indirect effect = 0.221; CI (0.175, 0.279)] (Fig. 1). In the second model, nightmares were significantly associated with SI (b = .109, SE = .031, *p* < .001). Similarly, depressive symptom severity was also found to significantly mediate the relationship between trauma-related nightmares and SI [bootstrapped unstandardized indirect effect = 0.136; CI (0.101, 0.177)] (Fig. 2).

## Discussion

The current study found that depressive symptom severity significantly mediated the relationship between sleep disturbances and SI, as well as the relationship between trauma-related nightmares and SI, in a sample of mental health treatment-seeking CAF members and veterans. These findings suggest that MDD should be treated as an independent risk factor for SI.

More than two thirds of our treatment-seeking sample of CAF members and veterans reported being at least moderately bothered by sleep disturbances; and almost 90% reported being at least moderately bothered by trauma-related nightmares. Congruent with previous research, these findings emphasize that sleep difficulties are a common concern for military personnel and veterans [42, 43], and echo the recent findings of Creamer and colleagues [44], who found that trauma-related nightmares were common amongst a cohort of 500 active duty United States military personnel. Our results also revealed that although sleep disturbances and nightmares were significantly associated with SI on their own, these associations seem to be mediated by depressive symptom severity, which is consistent with previous research supporting an indirect role of sleep disturbance [4, 23]. However, unlike prior research [4], we did not find the association between trauma-related nightmares and SI to be mediated by PTSD.

Unfortunately, there are currently few evidence-based practices to prevent and treat sleep difficulties among military members [1]. One promising therapy is Cognitive Behavioural Therapy for Insomnia (CBT-I). Recently, Trockel and colleagues [45] found that CBT-I was associated with reductions in the odds of SI and depressive symptoms in their sample of veterans presenting for insomnia treatment. Further, a systematic review by Taylor and Pruiksma [46] found support for CBT-I as a viable psychotherapeutic modality for the treatment of comorbid insomnia and psychiatric disorders. Exposure, Relaxation, and Rescripting Therapy adapted for military members (ERRT-M) has also shown some initially promising results. In their small (*N* = 19) pilot study of trauma-exposed veterans, Bailliett and colleagues [47] found that improvements in nightmare frequency, depression severity, sleep quality, and insomnia severity were achieved one week into ERRT-M therapy, and maintained 2 months later.

Results should be interpreted with caution in light of the following limitations. We relied on cross-sectional data obtained from a treatment-seeking population composed primarily of CAF veterans. As such, we cannot evaluate causal relationships between sleep disturbances, nightmares, and SI; and results may not be generalizable to other military, veteran, or clinical populations. Single-item measures were used to assess SI, sleep disturbances, and trauma-related nightmares. The sleep disturbances item combines difficulty initiating sleep and staying asleep. It is plausible that there are unique challenges associated with difficulty falling vs. staying asleep. Similarly, there may be differences between disturbing dreams and nightmares that cause one to arouse from their sleep that were not captured as a result of the language used in this single item. Lastly, the item used to assess SI ideation does not clearly distinguish between suicidal thought and non-suicidal self-injury. Additionally, because these single-item indicators were removed from the PHQ-9 and PCL-M to control for inflation, we essentially examined a version of depressive symptom severity that did not include sleep disturbances and SI, and a version of PTSD symptom severity that did not include sleep disturbances and nightmares. Although this approach has been used in previous research [4], findings might not accurately depict the clinical presentation of depression and PTSD among military personnel and veterans.

## Conclusion

The results from the present study contribute to furthering our understanding of the relationship between sleep disturbances, trauma-related nightmares, and SI in the presence of other mental health diagnoses. Findings implicate depression symptom severity as the greatest risk factor for SI, emphasizing the importance of targeting depression to mitigate suicide risk within military personnel and veterans. Incorporating therapies that target sleep and trauma-related nightmares into the management of depression and PTSD may provide additional therapeutic benefit and, consequently, further diminish the risk of SI. Treatment of psychiatric comorbidities, such as depression and sleep disturbances, may be especially crucial in reducing risk of SI in treatment-seeking military populations, particularly since previous research shows that individuals with military related-PTSD may experience poorer treatment response compared to their civilian counterparts [6]. Continued research examining the relationship between sleep disturbances, nightmares, and SI within varying clinical populations, especially at the meta-analysis level, may shed additional light on the direct and indirect effects of sleep-related variables.

## Abbreviations

AUD: Alcohol use disorder
CAF: Canadian Armed Forces
CBT-I: Cognitive behavioural therapy for insomnia
CFMHS: Canadian Forces Mental Health Survey
DSM-IV: Diagnostic and Statistical Manual of the American Psychiatric Association, Fourth Edition
ERRT-M: Exposure, Relaxing, and Rescripting Therapy for military
GAD: Generalized anxiety disorder
MDD: Major depressive disorder
PCL-M: PTSD Checklist, Military version
PHQ: Patient Health Questionnaire
PTSD: Posttraumatic stress disorder
SI: Suicidal ideation
